# Migrative armadillo optimization enabled a one-dimensional quantum convolutional neural network for supply chain demand forecasting

**DOI:** 10.1371/journal.pone.0318851

**Published:** 2025-03-03

**Authors:** Mohamed Irhuma, Ahmad Alzubi, Tolga Öz, Kolawole Iyiola

**Affiliations:** Institute of Social Sciences, University of Mediterranean Karpasia, Mersin, Turkey; Thapar Institute of Engineering and Technology: Thapar Institute of Engineering and Technology (Deemed to be University), INDIA

## Abstract

Demand forecasting is a quite challenging task, which is sensitive to several factors such as endogenous and exogenous parameters. In the context of supply chain management, demand forecasting aids to optimize the resources effectively. In recent years, numerous methods were developed for Supply Chain (SC) demand forecasting, which posed several limitations related to inadequate handling of dynamic time series patterns and data requirement problems. Thus, this research proposes a Migrative Armadillo Optimization-enabled one-dimensional Quantum convolutional neural network (MiA + 1D-QNN) for effective demand forecasting. The Migrative Armadillo Optimization (MAO) algorithm effectively optimizes the hyperparameters of the model. Specifically, the 1D-QNN approach offers exponential speed in the forecasting tasks as well as provides accurate prediction. Furthermore, the K-nearest Neighbor imputation technique fills the missing values, which preserves the data integrity as well as reliability. The experimental outcomes attained using the proposed model achieved a correlation of 0.929, Mean Square Error (MSE) of 7.34, Mean Absolute Error of 1.76, and Root Mean Square Error (RMSE) of 2.71 for the supply chain analysis dataset. For DataCo smart SC for big data analysis dataset, the MiA + 1D-QNN model achieved the correlation of 0.957, Mean Square Error (MSE) of 6.00, Mean Absolute Error of 1.62, and Root Mean Square Error (RMSE) of 2.45.

## 1. Introduction

Demand forecasting is a significant task, which requires historical demand data as well as forecasting techniques to predict future demands [1]. Demand forecasting entails intricate processes, particularly when it comes to supply chain management (SCM). Because demand forecasting has a direct impact on production planning, it is therefore a crucial part of SC management. To maximize expenses and income, firms utilize predictive analytic approaches to forecast demand. However, because of underlying volatilities and unpredictable complexities, it is challenging to forecast demand with high precision [2]. The global manufacturing sector has seen growth constraints since the global financial crisis as a result of the prolonged economic downturn and growing labor and raw material costs. Because of declining birth rates and a preference for services over low-wage manufacturing, industrialized countries see a decline in the number of workers in the manufacturing sector and a shift in the industrial structure toward a service-oriented economy [3]. Therefore, meticulous manufacturing planning is needed. Optimizing manufacturing schedules is intimately linked to effective inventory management. While having too little inventory could make it difficult to satisfy consumer requests, having too much inventory could result in contamination or material waste. Consequently, businesses should use effective inventory management to maintain the proper quantity of inventory [4,5].

Since load forecasting necessitates matching generating units between supply and demand, it is essential to the design and operation of electric generators. Forecasting durations are classified into three categories: short-term, medium-term, and long-term [6]. Monthly forecasting is regarded as medium-term, and annual data forecasting is regarded as long-term. Short-term load forecasting, which includes daily load forecasting, spans periods from hours to weeks. The forecasting model’s accuracy has the potential to lower operational costs by eliminating the need for standby generators for spinning reverse. However, the characteristics of the input and the model fitting determine how reliable the projected results are [7]. Researchers have found several new external indicators in the last ten years that can be used to predict future product demand. The weather, economic data, social media on the internet, and sentiment indexes are shown to be the most significant determinants. There are two main categories into which many load-forecasting techniques fall: parametric and nonparametric. Linear regression and other mathematical and statistical equations serve as the foundation for parametric approaches.

Nonparametric techniques include Artificial Intelligence and Machine Learning (ML) based methods including expert systems, fuzzy logic, Deep neural networks (DNN), and Artificial Neural Networks (ANNs). In the past ten years, numerous hybrid combinations of neural networks with nature-inspired algorithms such as Genetic Algorithms (GA), Particle Swarm Optimization (PSO), and others. Numerous researchers have also shown that the very efficient models developed by these hybrid combinations of intelligent forecast methods were quite accurate and had a high degree of generality [8]. The success of this strategy depends on the availability of publicly available natural language data. Moreover, the method requires manual training of natural language processing classifiers to extract a corpus of text that illustrates the relationships within the SC [9]. A specific kind of neural network called a Graph Neural Network (GNN) is made to extract data from graph data structures, including SCs [10]. Although current technologies, such as track-and-trace RFID, aid in improving the overall visibility of data flow, they were not intended to directly address the issue of structural visibility because they were not made to identify the SC structure [9],^11^]. Federated learning is a distributed machine learning framework that uses a safe, privacy-preserving encryption method to enable decentralized participants to work together on machine learning model training while guaranteeing that their personal information stays in the local region. But it also brings with it certain drawbacks, like vulnerability to a central node, possible overhead in transmission, and difficulties managing huge amounts of data [12].

To tackle the above-mentioned limitations, this research designed a MiA + 1D-QNN model for SC demand forecasting. The utilization of the K-nearest Neighbor (KNN) imputation technique enhances data reliability and effectively handles the missing values. Moreover, the extraction of statistical features, sale frequency features, inverse sale frequency features, and retail features offers significant details of the input, thus enhancing the prediction ability of the proposed model. The MiA + 1D-QNN collectively enhances demand forecasting tasks. The primary contributions of this research are explained as follows:

Migrative Armadillo Optimization algorithm (MAO): Combining the characteristics of walrus and armadillo provides a synergistic approach that capitalizes on their strengths. This hybridization can lead to more robust and efficient optimization solutions, making it particularly useful for demand forecasting and other complex tasks. Moreover, the MAO algorithm is specifically designed to optimize the learning parameters of the model to attain significant performance.

Migrative Armadillo Optimization enabled One-dimensional Quantum convolutional neural network (MiA + 1D-QNN): The quantum computing principles leveraged in the MiA + 1D-QNN offer considerable improvements in the SC demand forecasting tasks and also minimize the overfitting issues. The combination of the MAO algorithm with the quantum CNN model speeds up the prediction process. By integration, the MAO algorithm optimizes the hyperparameters and also solves global optimization problems.

The following sections of the article are structured as follows, section 2 describes the literature review of the related works in the SC demand prediction task, and Section 3 explains the proposed methodology of the proposed model. The experimental evaluation of the MiA + 1D-QNN model is explained in section 4 and the research conclusion is discussed in section 5.

## 2. Literature review

The Recurrent Neural Network (RNN)-based demand prediction model was implemented by Yuseon Kim and Kyongseok Park [5] to identify the clustering technique. The model effectively predicted the actual demands from complex and dynamic environments with high robustness. In addition, the RNN model demonstrated high predictive accuracy. However, in the real-time outlier detection method, the performance of the approach was less efficient.

Pyae Pyae Phyo and Chawalit Jeenanunta [7] combined with Classification and Regression Tree (CART) as well as the Deep Belief Network (DBN) to enhance the accuracy of prediction tasks. Furthermore, the CART method provided a better insight classification rate other than manual classification (MC) and the DBN model forecasted the regular load demand. Moreover, compared to other advanced DL technologies, the model has limited performance.

An efficient demand-forecasting method was modeled by Myungsoo Kim *et al.* [3] based on Long Short-Term Memory (LSTM) and the 2D Kernal Density Estimation (2D-KDE) techniques, which predicted the demand cost with superior reliability. In addition, this approach enhanced the real-world applicability as well as minimized the computational cost. Despite that, the model can only be utilized in large-size enterprises.

Edward Elson Kosasih and Alexandra Brintrup [9] employed a Graph Neural Network (GNN) based automated technique for hidden link prediction in a SC. The GNN-based automated method informed the supply network re-design efforts while high-risk factors were detected through scenario planning. Nevertheless, an integrated gradient (IG) did not verify or explain the decision-making procedure of the algorithm which led to a challenge in all across ML tasks.

Sushil Punia and Sonali Shankar [1] combined LSTM and Random Forest (RF) model for demand forecasting which integrated real-time information in demand planning. In addition, this method served as a decision support system, which aided to forecasting and decision-making process easier. However, the demand forecasting method was only applicable to food production industries. Therefore, developing forecasts the production with other industries was a challenging task in this method.

Muhammad Yasir *et al.* [2] presented an ensemble model for demand forecasting. The model showed that the appreciation of the exchange rate boosted the exports of textile apparel among macro-level factors. Moreover, the model has poor performance due to limited time-series data.

Hexu Wang *et al.* [12] introduced the LSTM approach based on vertical federal learning (Fed-LSTM) for demand forecasting which solved the problems created in data security. Furthermore, the Fed-LSTM model can capture historical information as well as demonstrate superior performance when compared to other methods. However, the LSTM model increased the computational complexity.

An ensemble DNN method was provided by Badar ul Islam and Shams Forruque Ahmed [8] for demand forecasting. This method showed better forecasting accuracy and generalization which led to lower the computational cost. Nonetheless, the variation in load balancing affected the system performance.

Nafi Ahmed and Faria Farzana [13] suggested the Support Vector Machine (SVM), using the advanced methodology of linear regression was introduced for forecasting sporadic demand. The model provides a wide opportunity to forecast sporadic demand more precisely reducing the error level and indirectly minimizing the holding cost. However, the model cannot work with a small amount of data. Table 1 depicts the summarized analysis of the existing method used for supply chain demand forecasting.

**Table 1:** Summarized analysis of existing methods used for supply chain demand forecasting.

S.NO.	Author’s	Methods	Benefits	Limitations	Dataset	Achievement
1.	Kim and Kyongseok Park [5]	RNN-based demand prediction model	The model effectively predicted the actual demands from complex and dynamic environments with high robustness.	However, in the real-time outlier detection method, the performance of the approach was less efficient.	Sales Dataset	The accuracy improvement rate (AIR) is 100.9% to 152.4%. This shows the improvement range of Prediction Accuracy.
2.	Pyae Pyae Phyo and Chawalit Jeenanunta [7]	Classification and Regression Tree (CART) as well as the Deep Belief Network (DBN)	The CART method provided a better insight classification rate other than manual classification (MC) and the DBN model forecasted the regular load demand.	The model has limited performance, compared to other advanced DL technologies.	–	MAPE – 5.36%
3.	Myungsoo Kim et al. [3]	Long Short-Term Memory (LSTM) and the 2D Kernal Density Estimation (2D-KDE) techniques.	The model enhanced the real-world applicability as well as minimized the computational cost.	The model can only be utilized in large-size enterprises.	–	Probability – 98.57%
4.	Edward Elson Kosasih and Alexandra Brintrup [9]	Graph Neural Network (GNN)	The GNN-based automated method informed the supply network re-design efforts while high-risk factors were detected through scenario planning.	An integrated gradient (IG) did not verify or explain the decision-making procedure of the algorithm which led to a challenge in all across ML tasks.	–	–
5.	Sushil Punia and Sonali Shankar [1]	LSTM and Random Forest (RF) model	The model served as a decision support system, which aided to forecasting and decision-making process easier.	The demand forecasting method was only applicable to food production industries.	Retailer’s dataset	MAE – 1.7428 RMSE – 2.2741
6.	Muhammad Yasir et al. [2]	An ensemble model for demand forecasting	The model showed that the appreciation of the exchange rate boosted the exports of textile apparel among macro-level factors.	The model has poor performance due to limited time-series data.	Time Series dataset	MAE – 0.002 RMSE – 0.004
7.	Hexu Wang et al. [12]	LSTM approach based on vertical federal learning (Fed-LSTM)	The Fed-LSTM model can capture historical information as well as demonstrate superior performance when compared to other methods.	The LSTM model increased the computational complexity.	Alibaba Cloud dataset.	MSE – 0.303 RMSE – 0.693
8.	Badar ul Islam and Shams Forruque Ahmed [8]	An ensemble DNN Model	The model showed better forecasting accuracy and generalization which led to lower the computational cost.	The variation in load balancing affected the system performance.	–	MAPE – 1.01%
9.	Nafi Ahmed and Faria Farzana [13]	An artificial intelligence algorithm, Support Vector Machine (SVM), using advanced methodology of linear regression	The model provides a wide opportunity to forecast sporadic demand more precisely reducing the error level and indirectly minimizing the holding cost.	The model cannot work with a small amount of data.	Sales dataset	MAPE – 0.11% MSE – 8.29%

### 2.1. Challenges

- The unavailability of the time series data for SC demand forecasting limited the performance of ensemble models. Moreover, the incorporation of macro-level indicators affected the prediction accuracy [2].
- The generalization ability of the LSTM-RF model was limited and prone to overfitting problems [1].
- The real-time applicability of the RNN-based demand prediction model was limited and also increased system complexity [5].
- The GNN technique was prone to black box issues, which affected the interpretability of the model in demand forecasting tasks. The GNN technique also increased the data imbalance problems [9].

### 2.2. Problem statement

In SCM predicting future demands accurately was a challenging task, several deep learning (DL) and machine learning approaches were developed for demand forecasting. However, the traditional methods face several challenges that hinder their effectiveness. The dynamic nature of SC models affected the prediction performance of the traditional methods. Moreover, geopolitical events, behavior changes, and market competition also affected the forecasting models. Furthermore, the accuracy of forecasting tasks was compromised by data quality issues, lack of data availability issues, and data imbalance problems [9]. Therefore, to overcome these problems this research offers a MiA + 1D-QNN model, which effectively resolves the above-mentioned limitations and enhances the SC demand forecasting task. The principle of quantum computing leveraged in the MiA + 1D-QNN model can process huge amounts of data with high accuracy and efficiency. This is particularly beneficial in supply chain management, where predicting demand accurately is crucial for optimizing inventory levels, reducing costs, and improving customer satisfaction.

## 3. Proposed migrative armadillo optimization-enabled one-dimensional quantum convolutional neural network for supply chain demand forecasting

**Fig 1:**
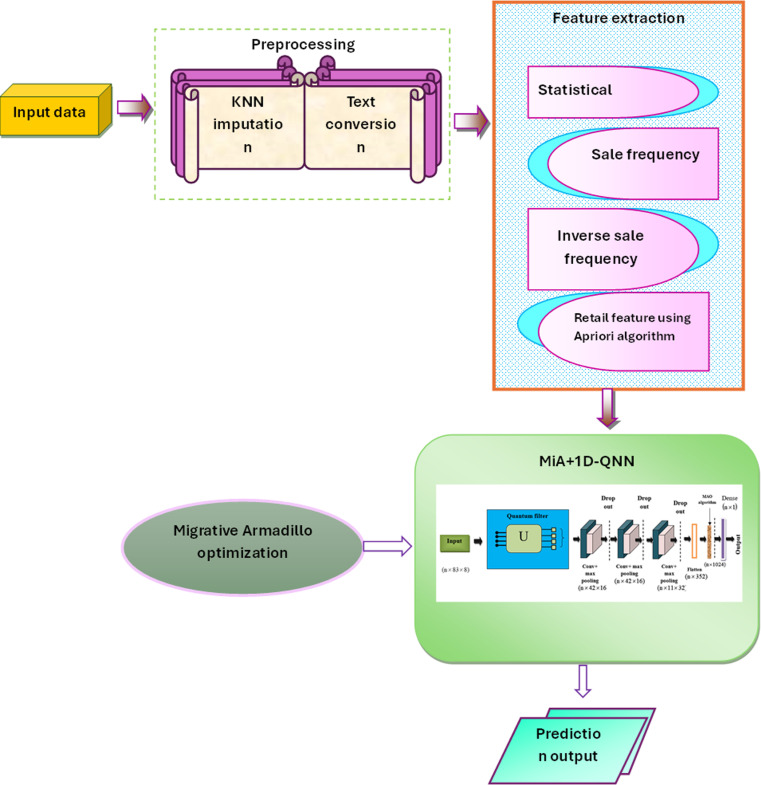
Proposed framework for supply chain demand forecasting.

The objective of this research is to predict the SC demands using the MiA + 1D-QNN model. Initially, the SC data is collected from the SC Analysis [14] and DataCo Smart SC for Big Data Analysis [15] Dataset. Before delving into the analytical process, the data undergoes a crucial pre-processing phase, which involves cleaning and organizing to ensure accuracy and consistency. Missing values are imputed using the KNN imputation algorithm. Following pre-processing, several techniques are employed to extract useful features from the dataset. Statistical feature calculation involves computing descriptive statistics and summarizing the distribution of numerical variables. Sale frequency analysis examines patterns over time or space to identify recurring trends or anomalies. Inverse sale frequency analysis investigates the frequency of sales of particular products or services concerning others. Retail feature extraction using the Apriori algorithm identifies association rules between different items in transactional data. Subsequently, the MAO algorithm is enabled in this research to optimize the learning parameters of the model. The MiA + 1D-QNN model combines principles from quantum computing with traditional neural network architectures. This fusion allows for more sophisticated analysis and processing of complex datasets by involving the principles of quantum mechanics. The Quantum CNN model generates outputs that include test data for evaluating the model’s performance and prediction models for estimating prices and costs. Fig 1 shows the proposed Framework for SC Demand Forecasting.

### 3.1. Input data

In this research, the raw data from SC Analysis [14] and DataCo Smart SC for Big Data Analysis [15] serve as input sources for demand forecasting tasks. The input data $\left\{d_{1},d_{2},.........d_{j}....d_{n}\right\}$ in the dataset *S* can be termed as


$$S=\left\{d_{1},d_{2},.........d_{j}....d_{n}\right\}$$ (1)

### 3.2. Imputation and text conversion based preprocessing

The input data for demand forecasting tasks includes several missing values and strings, which can reduce the statistical power of the model. Therefore, preprocessing is crucial for this task. Here, the preprocessing technique such as KNN imputation and text conversion is performed to enhance the reliability and precision of the proposed approach.

Missing data is a significant issue in demand forecasting tasks, which can be solved using imputation techniques. Imputation is performed by removing the unwanted data from the dataset and replacing the missing values with suitable alternatives. Several imputation techniques have been developed in recent times to handle missing values. However, the statistical and mean imputation techniques provide misleading information and reduce the accuracy of the model. Therefore, this research utilized a (KNN) [16] imputation technique. The KNN imputation algorithm employs just similar examples with the incomplete pattern, rather than using all occurrences in the data. The KNN imputation technique replaces missing observations in a dataset with values from records that are similar to the missing observations. Typically, an Euclidean distance function is used to determine the similarity [17]. The KNN establishes a collection of k nearest neighbors before using the average of its neighbors’ observed values to replace any missing observations for a particular variable. The distance $I\left(d_{i},d_{j}\right)$ between the observations with missing data $d_{i}$ and without missing data $d_{j}$ can be described using the following equation


$$I\left(d_{i},d_{j}\right)=\sqrt{\sum_{a=1}^{N}\left(d_{ia}-d_{ja}\right)}$$ (2)

Based on the smallest distance value the closest observation of the k value is determined, which can be mathematically evaluated as


$$\omega_{k}=\frac{1}{I{\left(d_{i},d_{j}\right)}^{2}}$$ (3)

Furthermore, the data imputation is carried out by calculating weight mean estimation ${\tilde{d}}_{a}$ that is described as


$${\tilde{d}}_{a}=\frac{\sum_{k=1}^{M}\omega_{k}m_{k}}{\sum_{k=1}^{M}\omega_{k}}$$ (4)

where $\omega_{k}$ denotes closest neighbor observations, $m_{k}$ indicates the attributes which contain missing data with parameter *k*. The KNN imputation technique is beneficial for this research because it can be used for both continuous and discrete variables.

Text conversion is the process of transforming input texts into a machine-readable format, particularly suitable for ML and DL approaches. In the context of SC data, which may contain strings that adversely affect system performance in demand forecasting tasks, it becomes crucial to convert these strings into numerical values. Here, the preprocessed data $d_{j}^{*}$ with the dimension of $\left(n\times 221\right)$is provided in the subsequent feature extraction phase. Where*n* indicates the number of input

### 3.3. Feature extraction using statistical sale and retail features

Feature extraction is a significant task for demand forecasting in SCs, in this research, the statistical features, sale frequency features, inverse sale frequency features, and retail features are extracted from the preprocessed data.

The statistical features provide valuable insights into the characteristics of data distributions, in this research the following statistical features are extracted from the preprocessed data. The mean is defined as the sum of all values in a dataset divided by the total number of values, which indicates the central tendency of the data [18]. Mathematically, the mean $\left(\mu \right)$ can be calculated as follows,


$$\mu =\frac{\sum_{j=1}^{n}d_{j}^{*}}{S}$$ (5)

The average of the squared difference between the data points is evaluated using variance. Variance $\left(Var\right)$ is mathematically computed as


$$Var=\frac{\sum_{j=1}^{n}{\left(d_{j}^{*}-\mu \right)}^{2}}{S}$$ (6)

The spread of data points around the mean is evaluated using standard deviation $\left(SD\right)$, which is mathematically formulated as


$$SD=\sqrt{\frac{\sum_{j=1}^{n}{\left(d_{j}^{*}-\mu \right)}^{2}}{S}}$$ (7)

The median $\left(Mid\right)$ is the center value in an ordered dataset. For an even number of values, the median is the average of the two middle values, while for odd numbers the center value is represented as the median.

Skewness $\left(\xi \right)$ measures the asymmetry of a distribution, which can be mathematically indicated as


$$\xi =\frac{\sum_{i=1}^{n}\left(d_{j}^{*}-\mu \right)}{\left(S-1\right)\times SD^{3}}$$ (8)

The flatness of the peakedness of a distribution can be measured using kurtosis $\left(kur\right)$. High kurtosis indicates more data concentrated around the mean, while low kurtosis indicates a flatter distribution [19].


kur=1n∑i=1ndj*−μ41n∑i=1ndj*−μ22 (9)

Entropy $\left(E\right)$ quantifies the uncertainty or randomness in a dataset, which can be mathematically denoted as


$$E=-sum\left(b*\log\left(b\right)\right)$$ (10)

Where *b* indicates the probability vector. The mode $\left(M\right)$ is the most frequently occurring value in a dataset. The geometric mean $\left(\mu_{G}\right)$ is the $n^{th}$ root of the product of *n* positive values, which is useful for calculating average growth rates. The smallest value in the dataset is known as minimum $\left(M_{\max}\right)$ and the maximum $\left(M_{\min}\right)$ is the largest value in the dataset. The sum $\left(\delta \right)$ is the total of all values in a dataset. The minimum $\left(\delta_{\min}\right)$ and maximum sum $\left(\delta_{\max}\right)$ can be calculated by subtracting the sum of the dataset from the minimum or maximum value, respectively. The extracted features are concatenated as follows $f_{st}=\left\|\mu \right\|Var\left\|SD\right\|Mid\left\|\xi \right\|kur\left\|E\right\|M\left\|\mu_{G}\right\|M_{\max}\left\|M_{\min}\right\|\delta_{\max}\left\|\delta_{\min}\right\|$, which has the feature dimension of $\left(n\times 36\right)$is further provided in the prediction model.

Sale frequency [20] refers to the number of sales transactions made over a specified period, which can be mathematically termed as


$$SF=\frac{O_{n}}{C_{n}}$$ (11)

where $O_{n}$ indicates the number of sales transactions and $C_{n}$ indicates the Total stocks, here $O_{n}C_{n}\in d_{j}^{*}$. Sale frequency usually has a positive relationship with several factors such as sales volume, perceived service quality, customer satisfaction, and balancing act. Sales frequency plays a crucial role in optimizing sales efforts and maintaining customer relationships. Inverse sale frequency represents the proportion of unsold stocks (inventory) relative to the total available stocks, which can be mathematically formulated as


$$I_{SF}=\frac{C_{n}-O_{n}}{C_{n}}$$ (12)

A high inverse sale frequency indicates excess inventory or slow-moving items, while a low inverse sale frequency suggests that most of the stocks are sold, which is greatly desirable. Monitoring inverse sale frequency helps optimize stock levels and improve SC performance. The extracted sale and inverse sale frequency have the dimension of$\left(n\times 22\right)$

Retail feature extraction involves identifying relevant features from retail data that significantly impact demand prediction. The Apriori algorithm is a classic association rule mining technique used to discover frequent items in transactional data [21]. Using the Apriori method for association rule mining involves two steps. Locating every item set that appears frequently in the database is the first step. Creating association rules from the collection of items that appear frequently is the second phase. Also measures the strength of association between items in an item set. In the context of demand forecasting, the retail features are extracted using the Apriori algorithm which enhances the performance of the demand prediction model. The features extracted using this algorithm are termed as $\left(A_{u}\right)$. The total features such as statistical features, sale frequency, inverse sale frequency, and retail features are concatenated as follows, which have the feature dimension of $\left(n\times 83\times 8\right)$and are subsequently transferred into the MiA + 1D-QNN for demand forecasting.


$$Z=\left\|f_{st}\right\|SF\left\|I_{SF}\right\|\left.A_{u}\right\|$$ (13)

Collectively the statistical sale and retail features offer superior performance for SC demand forecasting tasks and are also useful in understanding consumer behavior and improving retail performance.

### 3.4. One-dimensional quantum CNN for demand forecasting

**Fig 2:**
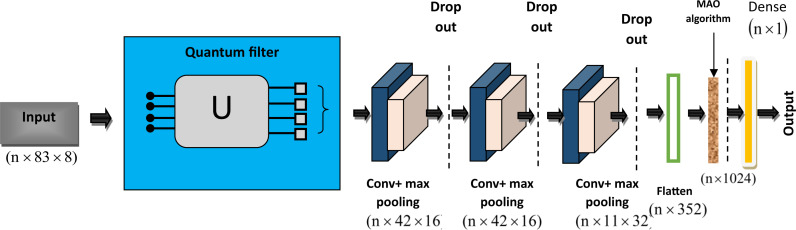
Architecture of MiA + 1D-QNN model.

Demand forecasting is a crucial problem in SC applications, in the current decade numerous techniques have been implemented for SC demand forecasting and inventory control. In this context, this research develops a 1D-QNN model that leverages quantum computing principles, potentially offering exponential speedup for demand forecasting tasks. The architecture of the MiA + 1D-QNN model is shown in Fig 2.

The traditional ML and DL approaches used for demand forecasting often exhibited various limitations. While the RNN model effectively processes sequence data that leads to improved prediction performance in various tasks. Because of their short-term memory problem, RNNs are less successful at identifying long-term dependencies in time series data [22]. During training, RNNs have trouble with vanishing or inflating gradients, which hinders their capacity to pick up complicated patterns. The Support vector Regression (SVR) can handle complex relationships between variables. However, it encountered local minimal problems or overfitting traps, which has led to poor predictions [13]. In complex SC networks, accurate supply forecasting is challenging due to cascading supply delays, dynamic node interactions, and resource availability. Therefore, to tackle these limitations, this research utilizes the Quantum Convolutional Neural Network (QCNN) model for effective prediction. Furthermore, the parameters of the models are tuned using the proposed MAO algorithm, which mitigates the local optimal and convergence problems.

The 1D classical convolutions and the 1D-QNN operate similarly. The primary distinction is in how the 1D quantum convolution carries out the operations. Subsections of one-dimensional signals are fed into the 1D quantum convolution, which uses variational Quantum Circuits (VQC) to produce a feature map in place of element-wise matrix multiplication operations. To extract the features, the VQC glides over the input tensor’s subsections [23]. A parameterized quantum circuit is applied after the considered portion has been encoded into an initialized quantum state.

**Fig 3:**
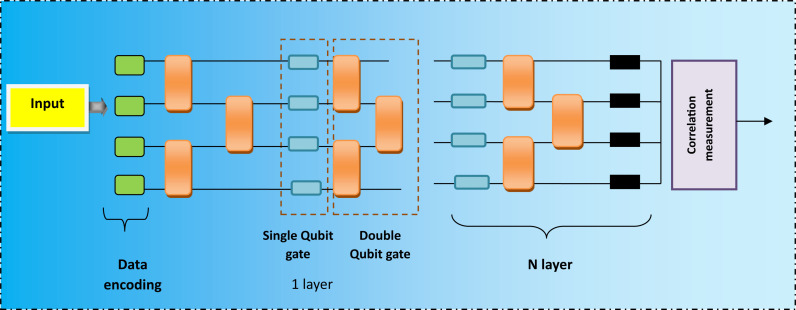
Quantum filter.


The data encoding of the quantum layer is performed using the following equation


$$\phi \left(Z\right)=\overset{N}{\underset{j=1}{⊗}}R_{y}\left(Z_{j}\right)$$ (14)

where  ⊗ indicates the tensor operator, $R_{y}$denotes the data encoding process. A quantum circuit can be expressed as a partial Fourier series, and the frequency spectrum can be expanded by repeating the encoding. This method may be appropriate for time series forecasting issues since the quantum circuit can be viewed as a Fourier series. The architecture of the Quantum filter is shown in Fig 3. The output produced from the quantum circuit is provided into the convolutional layer, which extracts important features from the previous layer. The output of the convolutional layer is expressed as

$$Y=\sigma \left(\sum W\phi \left(Z\right)+\zeta \right)$$ (15)

where *W*indicates the weights and *ζ*denotes the bias. Followed by the convolutional layer, the max pooling layer is employed, which decreases the dimensionality of the data and also minimizes system complexity. In this research, three convolutional and max pooling layers are employed. Followed by each max pooling layer drop out layer is utilized, which reduces overfitting issues. Furthermore, the flattened layer is utilized to convert the multi-dimensional data into a single dimension [24]. The dense layer with linear activation is used to perform prediction tasks effectively. As SC systems become increasingly complex, the ability to quickly adapt to new information and optimize accordingly makes MiA + 1D-QNN a powerful tool for demand forecasting tasks. The integration of quantum computing with CNN enables the model to focus on intricate features as well as the correlations within the data.

### 3.5. Migrative armadillo optimization

The MAO algorithm mimics the hunting strategy of armadillo [25] when moving towards the position of the prey and digging termite mounds as well as the migration behavior of walrus [26]. By combining the distinct behaviors, they create a balanced approach that enhances both exploration and exploitation. The MAO algorithm is suitable for solving global optimization problems. The combined algorithm offers promising solutions for SC demand forecasting tasks.

#### 3.5.1. Inspiration.

Walruses are Large, flip-flopped sea mammals, which are found sporadically in the subarctic and Arctic waters of the Northern Hemisphere, particularly near the North Pole. Walrus migration to rocky beaches or outcrops occurs in late summer as the weather warms and the ice melts. These moves are characterized by large, spectacular walrus aggregations. The social interactions and innate behaviors of walruses are examples of intelligent behavior. The key intelligent behaviors of walrus are: (i) direct individuals to eat by having the member with the longest tusks lead the way. (ii) Walrus migration to stony beaches (iii) Combat or flee from apex predators. The largest extant species of armadillo in jeopardy is the giant armadillo, which is found in South America. Among the gigantic armadillo’s instinctive habits, this animal’s approach when is considerably more common to attack termite mounds and then dig them to hunt and feast on termites. The two natural activities of gigantic armadillos during attacking, hunting, termite mounds, and excavating them to eat them have been mathematically modeled. The hunting technique of giant armadillos is by far the most noticeable of their natural actions. Therefore, combining the hunting strategy as well as escaping strategies can lead to better global search capabilities. The learning parameters*W*,*ζ* of the 1D-QCNN models are optimized using the MAO algorithm, which is performed in the eighteenth layer of the MiA + 1D-QNN model.

*Algorithm initialization***:** During initialization, values for the search space’s decision variables are determined for each solution based on where it is in the problem-solving space. Together, the solutions make up the algorithm’s population, which can be mathematically represented by a matrix in Eq 16. Eq 17 is used to randomly set the primary position of each solution in the search space at the start of the process.


$$G={\left[\begin{array}{l} G_{1}\\ \vdots \\ G_{i}\\ \vdots \\ G_{N} \end{array}\right]}_{N\times m}$$ (16)

where $G_{i}$ indicates $i^{th}$ solution, $G_{N}$ denotes the $N^{th}$ number of solutions, and *m* indicates the number of decision variables.


$$G_{i}^{t}=L+r_{1}\left(U-L\right)$$ (17)

where $G_{i}^{t}$ represents the position of $i^{th}$ solution at $t^{th}$ iteration, *U* and *L* signifies upper and lower bounds, $r_{1}$ signifies the initial kinetic movement factor $\in \left(0,1\right)$.

*Fitness evaluation***:** The algorithm’s objective function is a pivotal component, which improves the convergence performance of the model. In the MAO algorithm, the fitness function is evaluated in terms of maximum accuracy, which is represented as follows


$$F\left(G_{i}^{t}\right)=\max\left(accuracy\left(G_{i}^{t}\right)\right)$$ (18)

*Phase (i): Look Up phase:* $\left|B_{t}\right|\geq 1$ In this phase, $\left|B_{t}\right|\geq 1$ denotes that the target is not in the search space or problem-solving space. It updates its position based on the sensing factor occurring nearby to the target, but it does not have the concentration of the sensing factor. This may be due to the search being done as an individual. For each solution, the location of other solutions that have a superior objective function is considered as better. Along with that, the solution includes the leading behavior which guides the other solutions in the group to find the target. Thus, the combination of leading group behavior along with the sense-based path-finding character of the solution emerges as a new one, which enhances the exploration power in the global search. Thus the mathematical expression for the hybrid character-based new position update is formulated as follows


$$G_{i}^{t+1}=0.5\left(G_{i}^{t}+r_{2}\left(R_{p}-JG_{i}^{t}\right)\right)+0.5\left(G_{i}^{t}+r_{3}\left(G_{R}-IG_{i}^{t}\right)\right)$$ (19)

where $r_{2}$ denotes the sensing factor for finding paths effectively, $R_{p}$ denotes the selected search space, $r_{3}$ signifies the group leading behavior to improve the solution’s position, $G_{R}$ indicates the strongest or fittest solution in the group, *I* denotes the learning factor, $\left|B_{t}\right|$ shows the sensing factor to determine the prey’s location in search space, the collective fitness factor of the group with respect to change in displacement is termed as *J*


$$J=\frac{\sum_{i=1}^{n}\left|F\left(G_{i}^{t}\right)\right|}{\left|G_{t-1}-G_{t}\right|}$$ (20)

The below equation denotes the improvement in the individual solution’s position based on the best solution’s position on leading behavior


$$G_{i}^{}= \{\begin{array}{l} G_{i}^{t+1},\;when\;F\left(G_{i}^{t+1}\right)>F\left(G_{i}^{t}\right)\\ G_{i}^{t},\;else \end{array}$$ (21)

where $F\left(G_{i}^{t}\right)$ indicates the fitness function,

*Phase (ii): Migrative Escape or attacking phase***:** $\left|B_{t}\right|<1$ The condition $\left|B_{t}\right|<1$ shows that the target is within the search space, which is achieved based on the strong odour concentration of the target, and also the solution has the natural ability to find the enemies within the search space. Thus, the solution uses the strategy of escaping and fighting with those enemies, which leads to a change in the position of the solution. Simulating this behavior improves the exploitation power in the local search in the search space around the other solutions. The migration behavior along with the spading skills to escape from the enemies and the migration along with aggressive attacking behavior is mathematically expressed in the below equation.

***Subcase (i): Escaping behavior:*** If $F\left(G_{en}\right)\geq F\left(G_{i}^{t}\right)$: When the fitness function of the attacking solution is greater than the fitness of the current solution, then the solution performs the escaping strategy. The mathematical formulation of the escaping behavior is computed based on the current solution, altering factor, and the location of the selected solution, which is represented as follows,


$$G_{i}^{t+1}=0.5\left(G_{i}^{t}+r_{4}\left(G_{K}^{t}-J.G_{i}^{t}\right)\right)+0.5\left(G_{i}^{t}+\left(1-2r_{5}\right)\frac{U-L}{t}\right)$$ (22)

where $G_{en}$ indicates the attacking solution, $G_{K}^{t}$ denotes the $K^{th}$ solution which is in migration, and the fitness function of the attacking solution is denoted as $F\left(G_{en}\right)$, and $\;r_{4}$ represents the factor that alters the position of the solution.

*Sub Case (ii): Attacking behavior:* $F\left(G_{en}\right)<F\left(G_{i}^{t}\right)$ This condition implies that the fitness function of the attacking solution is less than the fitness of the current solution. The attack behavior-based solution updation is performed using the local lower and local upper factors that are determined as follows,


$$G_{i}^{t+1}=0.5\left(G_{i}^{t}+\left(L_{loc}\left(U_{loc}-r_{6}L_{loc}\right)\right)\right)+0.5\left(G_{i}^{t}+\left(1-2r_{5}\right)\frac{U-L}{t}\right)$$ (23)

where $r_{5}$ represents the spade factor of the solution, *t*denotes the iteration, $r_{6}$indicates the random numbers, $L_{loc}$ and $U_{loc}$ indicates the local lower and local upper factors respectively $L_{loc}\to \frac{L}{t};U_{loc}\to \frac{U}{t}$. Thus, the solution makes a good balance between escaping and attacking strategies, which eventually improves the experience of the solution to make important decisions while it stuck at the local optimum levels.

**Fig 4:**
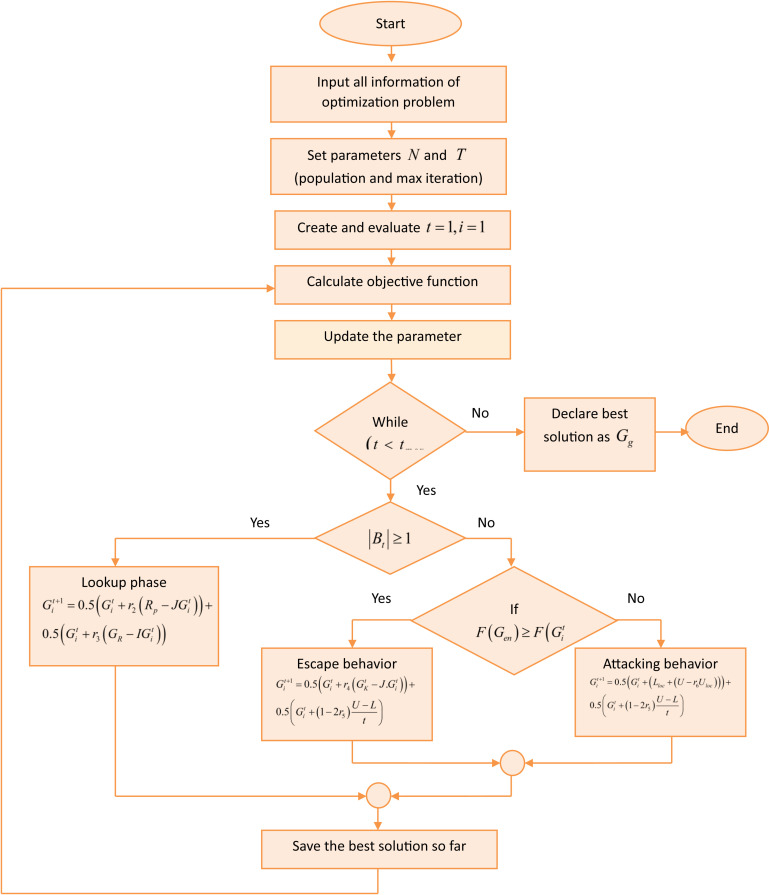
Flow diagram of the MAO algorithm.

*Termination***:** When the algorithm exceeds the limit of maximum iteration $t<t_{\max}$, it stops and updates the global best solution for improving the model’s performance. The flow diagram of the MAO algorithm is illustrated in Fig 4.

## 4. Results and discussion

The comparative and performance evaluation of the MiA + 1D-QNN model is discussed in this section. The analysis is performed in terms of the SC Analysis dataset and DataCo smart SC for the big data analysis Dataset. Moreover, the performance measures including MSE, RMSE, MAE, and correlation are established to accurately identify the forecasting ability of the MiA + 1D-QNN model.

### 4.1. Experimental setup

The research execution is conducted in “Python” software on a Windows 10 operating system equipped with 16 GB RAM. The parameters used in this research are a learning rate of 0.05, an Optimizer as ADAM, a batch size of 64, and an optimization population size is 100.

### 4.2. Dataset description

In this research, the SC Analysis dataset and DataCo smart SC for big data analysis Dataset are used for model training and testing.

#### 4.2.1. DataCo smart SC for big data analysis dataset [15].

The dataset comprises data related to Production, Sales, Provisioning, and Commercial Distribution. To generate knowledge, it also permits the connection of unstructured and organized data. The types of products are clothing, sports, and electronic supplies.

#### 4.2.2. SC analysis dataset [14].

It is the process of collecting, analyzing, and interpreting data related to the movement of products and services from suppliers to customers. The data is collected from fashion and beauty startups, which comprises product type, price, shipping times, order quantities, shipping costs, location, production volumes, and other parameters.

### 4.3. Performance metrics.

In this research, the MiA + 1D-QNN model’s effectiveness is analyzed using performance metrics such as MSE, correlation, Root Mean Squared Error (RMSE), and Mean Absolute Error (MAE).

### 4.4. Analysis based on supply chain analysis dataset

#### 4.4.1. Performance analysis.

**Fig 5:**
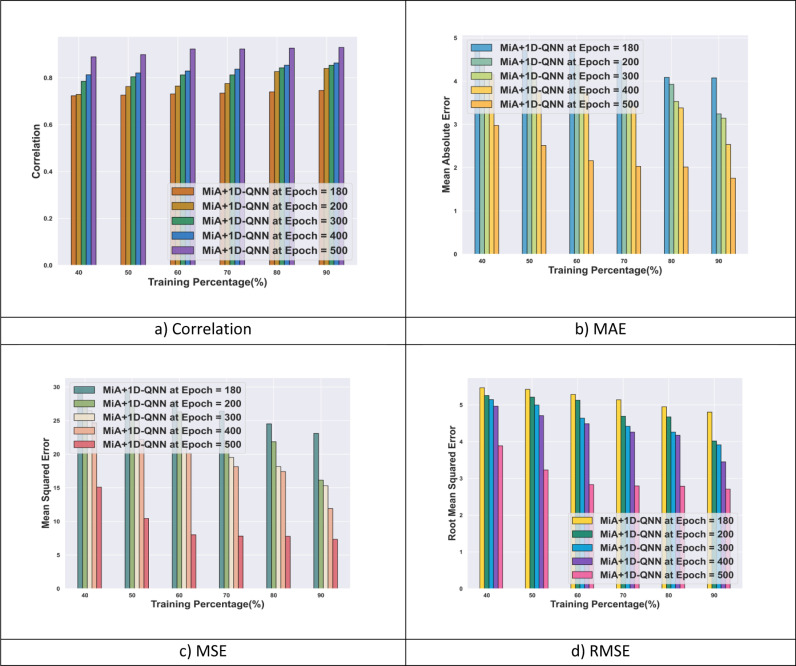
Performance analysis of MiA + 1D-QNN model with supply chain analysis dataset.

The MiA + 1D-QNN model’s performance evaluation with the Supply Chain Analysis dataset for SC demand forecasting is depicted in Fig 5. Regarding MAE, the MiA + 1D-QNN attains the value of 1.62 at TP 90 and epoch 500. For various epochs 100, 200, and 500 and TP 90, the MiA + 1D-QNN obtains MSE of 23.09, 16.15, and 7.34 respectively. The MiA + 1D-QNN model for SC demand forecasting obtains the RMSE of 2.71 for TP 90 and epoch 500. Moreover, in terms of correlation, the MiA + 1D-QNN model attains 0.93 concerning epoch 500 and TP 90. The use of 1D-QNN techniques enhances the predictive efficiency of this research in SC demand forecasting tasks.

#### 4.4.2. Comparative analysis.

**Fig 6:**
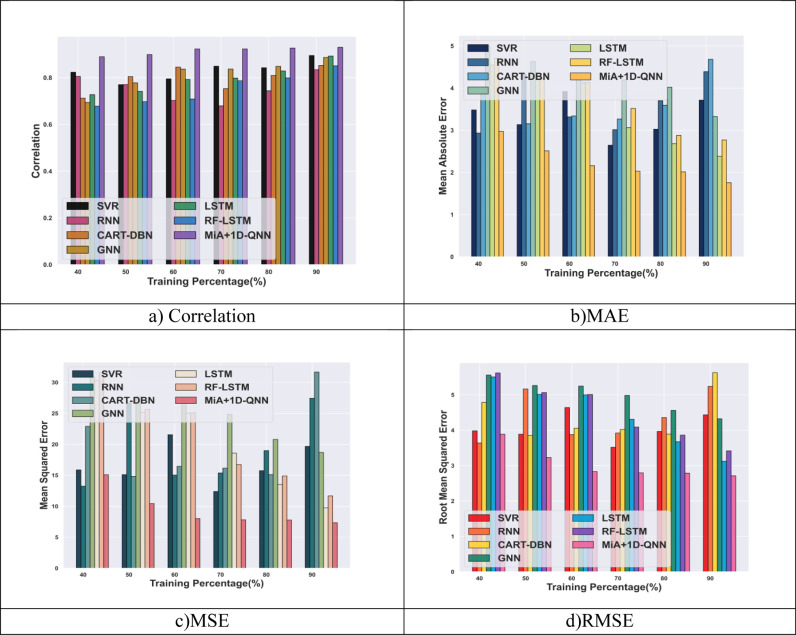
Comparative analysis of MiA + 1D-QNN model with supply chain analysis dataset.

The comparative evaluation of the MiA + 1D-QNN with other conventional techniques such as SVR [13], RNN [5], CART-DBN [7], GNN [9], LSTM [12], and RF-LSTM [1] in terms of RMSE, MSE, MAE, and correlation is illustrated in Fig 6. For TP 90, the MiA + 1D-QNN model attains a minimal MSE of 7.34, which is comparatively less than CART-DBN by 24.33, and SVR by 12.33. In terms of RMSE, the MiA + 1D-QNN gets a minimal value of 2.70, which is less than the other conventional technique. The correlation of the MiA + 1D-QNN model is 0.92, which shows enhancement over the existing LSTM by 4.01%, and GNN by 4.64%. Furthermore, for the MAE measure, the MiA + 1D-QNN model achieves a minimal value of 1.75, which is minimized over the established RNN by 2.63 and GNN by 1.56. Thus, the experimental results describe that the MiA + 1D-QNN model offers better performance than the conventional methods employed for demand forecasting. This significant performance is due to the utilization of the proposed QNN and MAO algorithm. Moreover, the multiple feature extraction techniques enabled in this research extract pertinent information from the input data, which leads to better prediction performance in SC demand forecasting tasks.

### 4.5. Analysis based on DataCo smart supply chain for big data analysis dataset

#### 4.5.1. Performance analysis.

**Fig 7:**
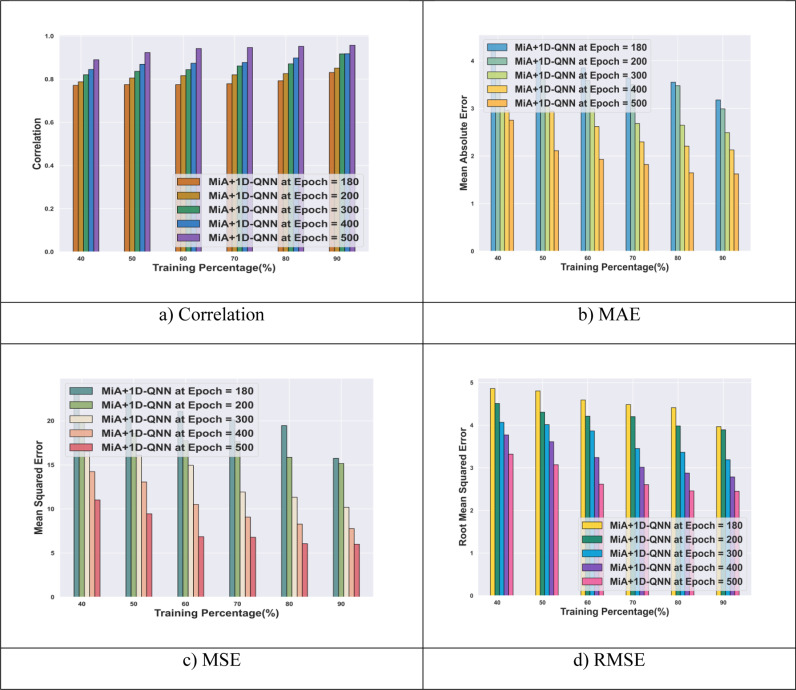
Performance analysis of MiA + 1D-QNN model with DataCo smart supply chain for big data analysis dataset.

Fig 7 illustrates the performance of the MiA + 1D-QNN model using the DataCo smart supply chain for big data analysis dataset for SC demand forecasting. The model achieves an MAE of 1.62 at TP 0f 90 and after 500 epochs of training. MSE results for the model at TP are 15.74, 15.15, and 5.99 for epochs 100, 200, and 500 respectively. Additionally, at TP 90 and epoch 500, the MiA + 1D-QNN model attains a RMSE of 2.45. The model also demonstrates a correlation of 0.95 at epoch 500 and TP 90. The use of the 1D-QNN technique in this research significantly improves the mode’s predictive accuracy with minimum error.

#### 4.5.2. Comparative analysis.

**Fig 8:**
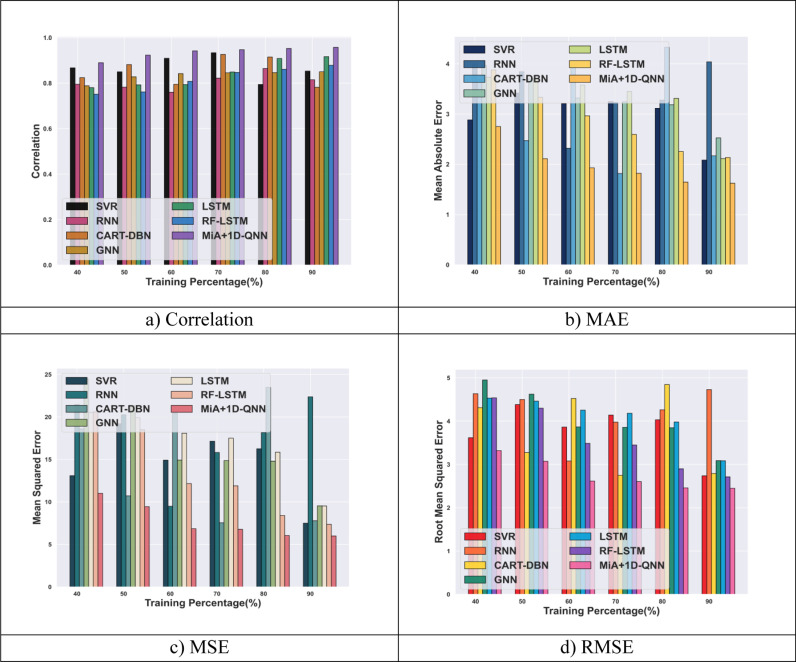
Comparative analysis of MiA + 1D-QNN model with DataCo smart supply chain for big data analysis dataset.

Fig 8 delineates the comparative evaluation of the MiA + 1D-QNN model with the conventional approaches concerning the metrics such as MAE, correlation, MSE, and RMSE for DataCo smart supply chain for big data analysis dataset. For TP 90, the MiA + 1D-QNN model attains a minimal MSE of 5.99, which is comparatively less than SVR by 1.51, and RNN by 16.37. In terms of RMSE, the MiA + 1D-QNN gets a minimal value of 2.44, which is less than the other conventional technique. The correlation of the MiA + 1D-QNN model is 0.95, which shows enhancement over the existing LSTM by 4.26%, and GNN by 11.22%. Furthermore, for the MAE measure, the MiA + 1D-QNN model achieves a minimal value of 1.62, which is minimized over the established RNN by 2.41 and GNN by 0.54. Thus, the experimental results describe that the MiA + 1D-QNN model offers better performance than the conventional methods employed for demand forecasting.

### 4.6. Ablation study

**Fig 9:**
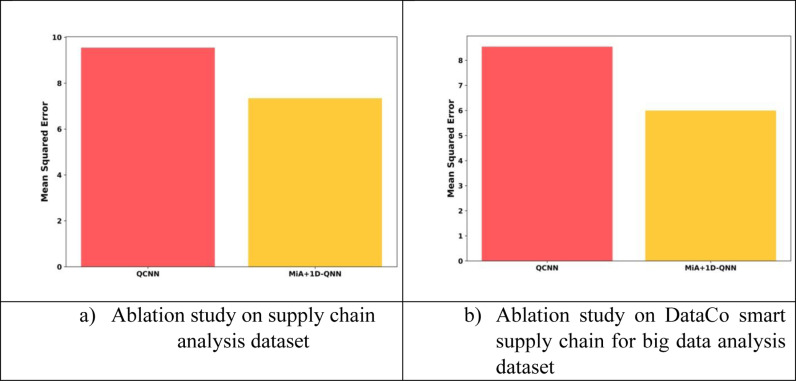
Ablation study of the MiA + 1D-QNN model.

Fig 9 depicts the ablation study of the MiA + 1D-QNN model on features in terms of MSE of both the supply chain analysis dataset and the DataCo smart supply chain for big data analysis dataset. For the supply chain analysis dataset, the QCNN attains an MSE of 9.54 and for the MiA + 1D-QNN model the MSE attained is 7.34. For the DataCo smart supply chain for big data analysis dataset, the QCNN attains an MSE of 8.54 and the proposed model attains a MSE of 6.00.

### 4.7. Computational complexity analysis

The computational time comparison between the MiA + 1D-QNN and the other existing methodologies is carried out to illustrate the efficiency of the proposed MiA + 1D-QNN method. By consistently requiring a lot less time than the other approaches, the results highlight the computational efficiency of the proposed MiA + 1D-QNN method. The recommended method has the shortest computation time of 20.55s when compared to the other existing methods. These results are shown in Table 2.

**Table 2:** Computational complexity analysis.

Methods	Computational Time (s)
SVR	20.81
RNN	20.68
CART-DBN	20.77
GNN	20.79
LSTM	20.80
RF-LSTM	20.81
MiA + 1D-QNN	20.55

### 4.8. Convergence analysis

**Fig 10:**
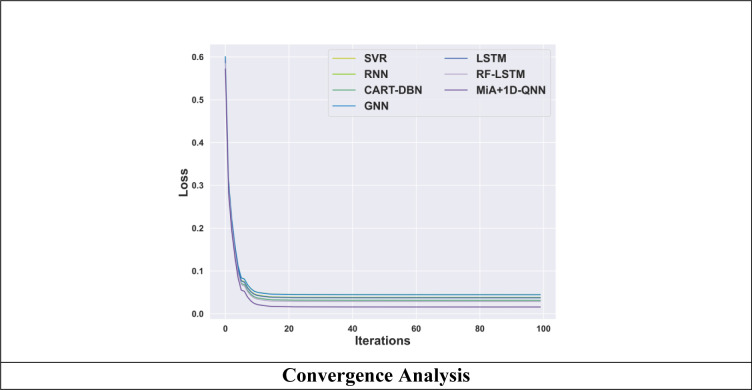
Convergence analysis of the MiA + 1D-QNN model.

The convergence analysis of the MiA + 1D-QNN model and other existing techniques is illustrated in Fig 10. The convergence rate comparison between the proposed MiA + 1D-QNN model and the other existing methodology is conducted across multiple to showcase the loss of the MiA + 1D-QNN model. The results highlighted the recommended strategy’s convergence rate because it consistently requires a significant reduction in error loss compared to other approaches. At 100 epochs, the suggested model has the lowest loss of 0.016 when compared to other existing approaches.

### 4.9. Comparative discussion

Table 3 describes the comparative discussion of the MiA + 1D-QNN model with the Supply Chain Analysis dataset and DataCo smart supply chain for the big data analysis dataset. The conventional approaches for the existing methods predictions have some limitations such as the SVR cannot work with a small amount of data. The CART-DBN method has limited performance; compared to other advanced DL technologies. The GNN method an integrated gradient (IG) failed to explain the decision-making procedure of the algorithm which led to a challenge in all ML tasks. In addition, the LSTM model was found to increase computational complexity. The RF-LSTM model was only applicable to food production industries. The traditional approaches established for SC demand forecasting have posed several limitations such as data availability, data imbalance problems, overfitting issues, and data quality problems. Therefore, this research proposes a MiA + 1D-QNN model, which addresses the above-mentioned limitations as well as enhances the system performance for demand forecasting tasks. The data imbalance problems in the existing methods are addressed by the KNN imputation techniques and this research utilizes two datasets for model training and testing, which resolves the data availability issues in the literature. Furthermore, the data quality problems are solved using multiple feature extraction techniques that enhance the prediction performance of the MiA + 1D-QNN. The proposed model attains a correlation of 0.929, with minimum MSE, RMSE, and MAE of 7.34, 2.71, and 1.76 respectively for the Supply Chain Analysis dataset with TP 90 and for DataCo smart supply chain for big data analysis dataset with TP 90 it attains correlation of 0.957, minimum MSE, RMSE, and MAE of 6.00, 2.45, and 1.62 respectively.

**Table 3:** Comparative discussion of MiA + 1D-QNN model for SC demand forecasting.

Methods/metrics		SVR	RNN	CART-DBN	GNN	LSTM	RF-LSTM	MiA + 1D-QNN
Supply Chain Analysis dataset	Correlation	0.894	0.834	0.851	0.886	0.892	0.850	0.929
	MSE	19.68	27.44	31.68	18.68	9.76	11.67	7.34
	MAE	3.71	4.39	4.68	3.33	2.38	2.77	1.76
	RMSE	4.44	5.24	5.63	4.32	3.12	3.42	2.71
DataCo smart supply chain for big data analysis dataset	Correlation	0.853	0.815	0.781	0.850	0.916	0.878	0.957
	MSE	7.50	22.37	7.79	9.54	9.52	7.36	6.00
	MAE	2.08	4.04	2.17	2.53	2.12	2.14	1.62
	RMSE	2.74	4.73	2.79	3.09	3.09	2.71	2.45

## 5. Conclusion

This research proposes the MiA + 1D-QNN model for demand forecasting in SC, which leverages the benefits of the quantum CNN model as well as the MAO algorithm. The active nature of Supply Chain models affected the prediction performance of the conventional methods. Data quality issues, lack of data availability, and data imbalance compromised the accuracy of forecasting tasks. Therefore, this research offers a MiA + 1D-QNN model to overcome these problems. This model effectively resolves the above-mentioned limitations and enhances the SC demand forecasting task. Furthermore, the utilization of quantum concepts in the proposed model processes huge datasets much faster than the traditional models, which provides timely and accurate forecasting results. The MiA + 1D-QNN can effectively identify dynamic patterns in the data, leading to more accurate predictions as well as enhancing the reliability of the model. The utilization of the MAO algorithm effectively optimizes the model’s learning parameters. The Apriori algorithm used for feature extraction obtains pertinent retail features that improve the model’s generalization ability. Furthermore, the experimental results of the proposed technique are compared with the other implemented approaches and the results exhibit better performance than the other established methods. The proposed MiA + 1D-QNN model attains a correlation of 0.929, minimum MSE, RMSE, and MAE of 7.34, 2.71, and 1.76 respectively for the Supply Chain Analysis dataset with TP 90. Meanwhile, the MiA + 1D-QNN model achieves a correlation of 0.957, minimum MSE, RMSE, and MAE of 6.00, 2.45, and 1.62 respectively for DataCo smart supply chain for big data analysis dataset with TP 90. However, the model is less efficient in complex data, it requires more feature extraction process to extract the relevant feature is one of the limitations of this research. In the future, the research will be designing an ensemble optimization technique with quantum learning approaches for an effective prediction of demand forecasting.
